# Supplementary material for: Usefulness of the Novel Snare-over-the-Guidewire Method for Transpapillary Plastic Stent Replacement (with Video)
Source: J Clin Med. 2021 Jun 28;10(13):2858. doi: 10.3390/jcm10132858 (PMC8268235; doi:10.3390/jcm10132858)
Supplement: Supplementary file 1 [file jcm-10-02858-s001.zip › jcm-1272142-SI.pdf]

Supplemental Table 1: Comparison of procedural success rates between the SOG and SOS groups.

	SOG group	SOS group	P-value
	Overall		
	(n=61)	(n=183)	
Success rate of removing the stent after inserting the guidewire into the bile duct where the stent was placed (%)	90.2 (55/61)	77.1 (141/183)	0.026
	Malignant		
	(n=31)	(n=82)	
Success rate of removing the stent after inserting the guidewire into the bile duct where the stent was placed (%)	93.6 (29/31)	81.7 (67/82)	0.147
	Benign		
	(n=30)	(n=101)	
Success rate of removing the stent after inserting the guidewire into the bile duct where the stent was placed (%)	86.7 (26/30)	73.3 (74/101)	0.150

$P<0.05$ was considered statistically significant.
SOG, snare-over-the-guidewire method; SOS, side-of-stent method; IQR, interquartile range

Supplemental Table 1 shows the results of comparing the procedure success rates between the SOG and SOS groups focusing malignant or benign. In both malignant and benign diseases, the success rate of the procedure was superior in the SOG group.
